# The reality of advanced airway management during out of hospital cardiac arrest; why did paramedics deviate from their allocated airway management strategy during the AIRWAYS-2 randomised trial?

**DOI:** 10.1016/j.resplu.2023.100365

**Published:** 2023-02-18

**Authors:** K. Kirby, M. Lazaroo, J. Green, H. Hall, R. Pilbery, G.A. Whitley, S. Voss, J. Benger

**Affiliations:** a University of the West of England, Bristol, United Kingdom; b South Western Ambulance Service NHS Foundation Trust, Exeter, United Kingdom; c University of Bristol, Bristol, United Kingdom; d University of Plymouth, Plymouth, United Kingdom; e James Paget University Hospital NHS Foundation Trust, Yarmouth, United Kingdom; f Yorkshire Ambulance Service NHS Trust, Wakefield, United Kingdom; g University of Lincoln, Lincoln, United Kingdom

**Keywords:** Emergency medical services, Out-of-hospital cardiac arrest, Airway management, Paramedic, Resuscitation, Advanced life support

## Abstract

**Background:**

AIRWAYS-2 was a large multi-centre cluster randomised controlled trial investigating the effect on functional outcome of a supraglottic airway device (i-gel) versus tracheal intubation (TI) as the initial advanced airway during out-of-hospital cardiac arrest. We aimed to understand why paramedics deviated from their allocated airway management algorithm during AIRWAYS-2.

**Methods:**

This study employed a pragmatic sequential explanatory design utilising retrospective study data collected during the AIRWAYS-2 trial. Airway algorithm deviation data were analysed to categorise and quantify the reasons why paramedics did not follow their allocated strategy of airway management during AIRWAYS-2. Recorded free text entries provided additional context to the paramedic decision-making related to each category identified.

**Results:**

In 680 (11.7%) of 5800 patients the study paramedic did not follow their allocated airway management algorithm. There was a higher percentage of deviations in the TI group (399/2707; 14.7%) compared to the i-gel group (281/3088; 9.1%). The predominant reason for a paramedic not following their allocated airway management strategy was airway obstruction, occurring more commonly in the i-gel group (109/281; 38.7%) versus (50/399; 12.5%) in the TI group.

**Conclusion:**

There was a higher proportion of deviations from the allocated airway management algorithm in the TI group (399; 14.7%) compared to the i-gel group (281; 9.1%). The most frequent reason for deviating from the allocated airway management algorithm in AIRWAYS-2 was obstruction of the patient’s airway by fluid. This occurred in both groups of the AIRWAYS-2 trial, but was more frequent in the i-gel group.

## Introduction

The AIRWAYS-2 randomised controlled trial (RCT) investigated the effect on functional outcome of a supraglottic airway device (i-gel) versus tracheal intubation (TI) as the initial advanced airway management (AAM) strategy during OHCA^1^. The majority of research investigating paramedic AAM in OHCA originates from observational studies with the risk of confounding factors^2^. Additional data from the AIRWAYS-2 RCT allows a detailed investigation of paramedic AAM during OHCA and an opportunity to further understand the AAM challenges encountered in the field.

Previously published literature has highlighted the difficulties confronted when performing AAM in out-of-hospital care. Including: the negative impact of an unfavourable laryngoscopic view^3^; bodily fluids obstructing the view of the larynx^3^, ^4^; patient obesity^3^; patient positioning^3^; traumatic injuries to the spine or face^3^, ^4^ and; limited access to the patient’s airway^4^. A systematic review completed in 2021^2^ found that although a supraglottic airways (SGA) is faster to insert, with a higher first pass success rate, aspiration may be more common when using SGA over tracheal intubation (TI). During the AIRWAYS-2 trial paramedics did not always follow their allocated airway management trial algorithm and this airway management algorithm deviation data gives important context to the complications that paramedics face when performing AAM during OHCA.

The aim of this study was to understand why paramedics deviated from their allocated airway management algorithm during the AIRWAYS-2 RCT, and to gain insights into paramedic decision-making and airway management during OHCA. This work has the potential to inform future clinical guidelines, training and research.

## Methods

AIRWAYS-2 took place in four Emergency Medical Services (EMS) providers (NHS ambulance services in England) between June 2015 and August 2017. The design of AIRWAYS-2, the clinical outcomes and relative cost effectiveness of the two strategies, along with the experiences of EMS staff (paramedics) taking part, have been published previously 1, 4, 5, 6, 7, 8. AIRWAYS-2 included patients who were aged 18 years or older, who had suffered a non-traumatic OHCA and who were treated by an AIRWAYS-2 study paramedic.

During the AIRWAYS-2 Study, paramedics were instructed to follow an airway management algorithm that required them to make two attempts at placing the randomly allocated airway device (Supplementary Figure 1). If both these attempts proved unsuccessful, then the paramedic could proceed to two attempts at placing the alternative device. If these two attempts were also unsuccessful, further management was at the paramedic’s clinical discretion. Prior to the start of AIRWAYS-2 the study team anticipated that paramedics would encounter challenges that would lead to occasional deviation from the allocated airway management algorithm. Paramedics were empowered to deviate from the trial airway management algorithm if they deemed it to be clinically necessary. The AIRWAYS-2 trial research team collected data from study paramedics concerning any algorithm deviations that occurred and the reasons for these. Data were entered electronically into a trial database.

We used a pragmatic sequential explanatory design utilising retrospective study data. Airway algorithm deviation data collected routinely during the AIRWAYS-2 trial were analysed in order to categorise and quantify the reasons why paramedics did not follow their allocated strategy of AAM during the trial. Data were included in the analysis if patients had received at least one attempt at AAM during the trial. An AAM attempt included where a study paramedic used an i-gel or a cuffed tracheal tube in an effort to manage the patient’s airway. In 2307/9248 (25 %) of patients included in AIRWAYS-2, airway management was handed over to another clinician. Patients where airway management was handed over to another clinician were excluded in order to focus analysis on the decision making of study paramedics.

Reasons for deviating from the AAM strategy were grouped into categories by two researchers, ML (Medical Statistician) and KK (Paramedic Senior Research Fellow). Categories were initially formulated by ML and then refined and finalised through consensus. Categories were quantified according to the study paramedic’s allocated airway management strategy and analysed using descriptive statistics. During AIRWAYS-2, reasons why study paramedics deviated from their allocated airway management were recorded as part of the study process. Free text entries collected from AIRWAYS-2 study paramedics by the research team provided additional context to paramedic decision-making in relation to each of the categories identified. One experienced qualitative researcher (KK) reviewed the qualitative data and selected data that was representative and added context and explanation to the quantitative data. Quotes were reviewed and agreed with the wider study team. The four EMS providers that participated in AIRWAYS-2 are represented by the letters A-D.

The sponsor for AIRWAYS-2 was South Western Ambulance Service NHS Foundation Trust. Research ethics approval was granted by the Oxford C-South Central Research Ethics Committee (reference 14/SC/1219) and Confidentiality Advisory Group approved the trial under Regulation 5 of the Health Service (Control of Patient Information) Regulations 2002. Trial Registration ISRCTN: 08256118.

## Results

During AIRWAYS-2, 9296 patients were enrolled. Of these, 4410 patients were allocated to TI and 4886 to the i-gel. In total, there were 5800 patients where AAM was attempted, and the patient was not handed over to another clinician. Of these 5800 patients, the study paramedic did not follow their allocated airway management algorithm in 680 (11.7 %) patients. There was a higher percentage of deviations in the TI group (399/2707; 14.7 %) compared to the i-gel group (281/3088; 9.1 %).

Table 1 reports the primary outcome of functional recovery of the main AIRWAYS-2 trial for those patients where the study paramedic followed and did not follow the allocated airway management algorithm. In patients where paramedics followed the allocated airway management algorithm a higher percentage of patients in the i-gel arm (97/2807; 3.5 %) achieved good functional recovery in comparison to the TI arm of the trial (44/2308; 1.9 %).

**Table 1:** Primary trial outcome for patients receiving at least one AAM attempt and who were not handed over to another clinician according to whether they followed the allocated airway management algorithm, by treatment group and overall.

		**Randomised to TI** **(n = 399)**		**Randomised to** **i-gel** **(n = 281)**		**Overall** **(n = 680)**	
Primary Outcome:							
	***Did not follow the allocated algorithm***						
	Good functional recovery (mRS 0–3 at 30 days/hospital discharge)	11/399	2.8 %	6/281	2.1 %	17/680	2.5 %
	***Followed allocated algorithm***						
	Good functional recovery (mRS 0–3 at 30 days/hospital discharge)	44/2308	1.9 %	97/2807	3.5 %	141/5115	2.8 %

mRS: modified Rankin Scale.

Table 2 categorises the reasons why paramedics did not follow their allocated airway management algorithm, and the number of patients in the TI and i-gel groups where this occurred.

**Table 2:** Reasons why paramedics did not follow their allocated airway management algorithm in the TI and i-gel groups.

	***Randomised to TI*** ***(n = 399)***		***Randomised to i-gel*** ***(n = 281)***		***Overall*** ***(n = 680)***	
***Reasons for not following algorithm***						
*Obstruction/blood/fluid in airway*	*50/399*	*12.5 %*	*109/281*	*38.7 %*	*159/680*	*23.4 %*
*Clinical decision*	*71/399*	*17.8 %*	*50/281*	*17.8 %*	*121/680*	*17.8 %*
*No reason given*	*44/399*	*11.0 %*	*38/281*	*13.5 %*	*82/680*	*12.1 %*
*Space/patient position issues*	*65/399*	*16.3 %*	*0/281*	*0 %*	*65/680*	*9.6 %*
*Equipment issues*	*23/399*	*5.8 %*	*31/281*	*11.0 %*	*54/680*	*7.9 %*
*Forgot to enrol*	*33/399*	*8.3 %*	*23/281*	*8.2 %*	*56/680*	*8.2 %*
*Patient’s anatomy*	*33/399*	*8.3 %*	*11/281*	*3.9 %*	*44/680*	*6.5 %*
*Solo responder*	*23/399*	*5.8 %*	*1/281*	*0.4 %*	*24/680*	*3.5 %*
*Futility*	*16/399*	*4.0 %*	*4/281*	*1.4 %*	*20/680*	*2.9 %*
*Improved patient condition*	*15/399*	*3.8 %*	*7/281*	*2.5 %*	*22/680*	*3.2 %*
*Poor airway view*	*20/399*	*5.0 %*	*0/281*	*0 %*	*20/680*	*2.9 %*
*Other*	*4/399*	*1.5 %*	*6/281*	*2.2 %*	*10/680*	*1.5 %*

Other includes do not attempt resuscitation; no end-tidal carbon dioxide monitoring and “other reason” documented by study paramedics.

### Obstruction/blood/fluid in airway

The most frequent reason for deviating from the allocated airway algorithm was recorded as an airway obstruction which included blood and fluid in the airway. Deviating for this reason occurred in both groups of the trial, but occurred more frequently in the i-gel group (50/399 12.5 % TI; 109/281 38.7 % i-gel). Free text data indicated that there is a preference for TI over i-gel when a patient’s airway is compromised by fluid, with a suggestion that the i-gel can become impractical when used in a patient with an airway that contains fluid.

Quote One: *“Clinical decision to intubate due to amount of fluid in airway” (C5734).*

Quote Two: *“i-gel first despite being on intubation group and part of a crew. 1. There was initially a lot of regurgitation, an OP[oropharyngeal airway] was ineffective and I attempted to ventilate with an i-gel whilst the intubation equipment was prepared. The patient was then successfully intubated. 2. The tube seemed to become displaced and on an attempt to re-intubate the laryngoscope blade failed to light up, so an i-gel was used again whilst a replacement was sourced. Once sourced the next attempt was successful“ (A19548).*

Quote Three: *“Only 1 attempt at i-gel I think it was properly sited but was spraying vomit across the trolly & clogging up so there didn’t see any point in reinserting it” (A18408)*.

### Clinical decision

The “clinical decision” category included a number of reasons for paramedics deviating from their allocated algorithm and occurred equally between groups (TI 71/399 17.8 %; i-gel 50/281 17.8 %). Decisions were made to support the patient’s best interests for the situation at the time.

Quote Four: *“After an intubation attempt swapped to OPA [an oropharyngeal airway] rather than another attempt with advanced equipment. Neither ETT/I-gel had worked well; thought best for patient” (A2763)*

Quote Six: *“Difficult scene to manage, clinical decision to use i-gel” (B8908).*

### Space/patient position issues

The category of space/patient position issues was limited to the TI group where 65/399 16.3 % of deviations from the allocated algorithm were for this reason.

Quote Seven: *“i-gel was used first as space was very tight on site” (A1832)*.

Quote Eight: *“Space position issues. Cardiac arrest on train. Used LMA [laryngeal mask airway]” (D9258)*.

### Equipment issues

Algorithm deviations in this category mostly concerned paramedics not having the correct equipment at their side and occurred more frequently in the i-gel group than the TI group (TI 23/399 5.8 %; i-gel 31/281 11.0 %). In addition, there were occasional equipment failures reported as illustrated in Quote 10.

Quote Nine: *“Job not passed as cardiac arrest, therefore i-gels not with paramedic” (D2283)*

Quote Ten: *“Failure of laryngoscope light meant swapped method” (A1178)*.

### Patient anatomy

Deviating because of the patient’s anatomy was more prominent in the TI group than the i-gel group (TI 33/399 8.3 %, i-gel 11/281 3.9 %).

Quote Eleven: *“Abnormal patient anatomy unable to ETT [endotracheal tube]/igel” B13969*.

Quote Twelve: *“One intubation attempt. Grade 4 view, very big neck, stuck to basics” (D591)*.

### Solo responder

In this category, deviations from the algorithm occurred predominantly in the intubation group OHCA (TI 23/399 5.8 %; i-gel 1/281 0.4 %).

Quote Thirteen: *“Had 3 attempts with i-gel before switching method. This was due to solo responder for long time, attempted 2 × size 4, while waiting wanted to go to size 5 i-gel just to check it wasn’t poor selection” A04108*.

Quote Fourteen: *“Unable to intubate due to being a solo responder and crew 20 minutes away” (D391)*.

### Futility

Paramedics occasionally deviated from the allocated algorithm where it became apparent that continued resuscitation would be futile (TI 16/399 4.0 %; i-gel 4/281 1.4 %).

Quote Fifteen: *“Intubation group of trial but only one i gel attempt. AIRWAYS-2 study paramedic believed situation futile” (A6243)*.

### Improved patient condition

Deviations from the allocated algorithm, where the patient had a return of spontaneous circulation (ROSC) or was making good respiratory effort occurred more frequently in the TI group of the trial (TI 15/399 3.8 %, i-gel 7/281 2.5 %).

Quote Sixteen: *“Making resp[iratory] effort on arrival, unable to tube” (B16471)*.

Quote Seventeen: *“ROSC before 2nd igel attempt”* (D14991).

### Poor airway view

This deviation was limited to the TI group of the trial and was documented where the study paramedic did not obtain an adequate view of the vocal cords during laryngoscopy (TI 20/399 5.0 %).

Quote Eighteen: *“Unable to visualise cords and confirm correct placement. Then patient had trismus” (B118)*.

## Discussion

The results of this analysis of airway management algorithm deviation during the AIRWAYS-2 trial provide valuable insights into paramedic experiences when providing AAM during OHCA. Paramedics were more likely to deviate from the allocated airway algorithm in the TI group than the i-gel group. The reasons recorded for paramedics deviating from the prescribed airway management algorithm included: airway obstruction, clinical decision, space and patient position problems, equipment issues, the patient’s anatomy, solo responding, futility, improving patient condition and poor airway view. Where paramedics followed the airway algorithm a higher percentage of patients survived with of good functional recovery in the i-gel arm in comparison to the TI arm of the trial.

The most frequent reason recorded for a deviation was an obstruction of blood or fluid in the patient’s airway, with this occurring more frequently in the i-gel group. Emesis has been reported in 32 % of OHCAs and is associated with reduced survival^9^. A soiled airway presents challenges unique to this setting. A case series^10^ of aspiration during in-hospital anaesthesia reported one case of the i-gel failing to protect the airway from aspiration. In the same case series, another two patients regurgitated and their airway was protected by the i-gel. The i-gel has been found previously to be less effective at preventing aspiration than a device with an inflatable cuff, and supraglottic airway devices are recognised as being less effective at preventing aspiration than TI^11^. Previous research by Voss and colleagues^12^ recognised that paramedics commonly change their airway choice because of regurgitation, however the authors could not determine whether regurgitation made ventilation impossible, or whether the paramedic was concerned about the risk of aspiration. This present study finds that paramedics make this decision based on the perceived risk of aspiration as well as failure to ventilate the patient.

Conversely, paramedics also made decisions to use the i-gel rather than TI when the patient’s airway was obstructed by fluid. A recent study investigating drug assisted intubation by anaesthetists using video laryngoscopy found that 77 % of failed first pass attempts were due to an airway obstructed by vomit, food, mucus or blood^13^. A soiled airway remains very challenging for paramedics to manage effectively in the out-of-hospital environment.

A “clinical decision” for deviating from the allocated algorithm incorporated a number of different scenarios where the paramedic adapted their management to the situation. This category reflects the challenging and dynamic nature of OHCA. Other reasons for deviating from the allocated algorithm were specific to the advanced airway, for example requiring space to perform tracheal intubation, space for an assistant and space for the equipment. In contrast, i-gel placement is generally easier than TI, and can be completed by a single operator with a median insertion time of 11 seconds in one study^14^. Conversely TI has been indicated to take a median time of five minutes during in-hospital cardiac arrest^15^, and this time would be expected to be longer out-of-hospital. One EMS provider (Service D) did not have i-gels as standard issue and paramedics in this service were allocated trial i-gels that the paramedic had to remember to take to an OHCA. This accounted for 29/31 (93.5 %) of the deviations from the algorithm for the equipment issues category in the i-gel group.

TI cannot be performed by a solo responder without interrupting lifesaving CPR whilst TI is taking place; therefore this deviation was permitted in the algorithm during AIRWAYS-2 and reflected in Supplementary Figure 1. Having a “poor (airway) view” was also limited to TI deviations because successful intubation requires direct visualisation of the vocal cords and passage of the tracheal tube^16^, ^17^. Deviation due to the patient’s anatomy was linked closely to “poor (airway) view.” There are known anatomical predictors for a difficult intubation including obesity, short neck, limited neck extension and large neck circumference^18^.

Patients were enrolled in the AIRWAYS-2 trial where they suffered a non-traumatic OHCA. In a proportion of the patients enrolled, prompt treatment such as early defibrillation was successful and resulted in a ROSC which impacted on the success and choice of AAM. Deviations due to “improving patient condition” occured more commonly in the TI arm and this is likely to reflect the fact that it takes longer to prepare for TI than i-gel. As a result of this time delay in attempting AAM in the TI group, the patient was more likely to achieve a ROSC and become less tolerant of AAM.

The strength of this study is that it explores the AAM decisions of paramedics taking part in a large multi-centre prospective trial of AAM in OHCA. This research provides detailed context regarding the AAM decisions that EMS clinicians (paramedics) make in practice. Limitations include reliance on study paramedic self-report regarding the reasons that they deviated from their allocated AAM strategy. No statistical tests were planned or completed. In addition, this study was limited to EMS providers in England and may not be generalisable to international EMS systems.

Following the publication of the primary results from AIRWAYS-2, TI was de-emphasised in the 2021 Resuscitation Guidelines,^19^ and it was suggested that only rescuers with a high TI success rate should perform TI. In many UK EMS services TI is now an enhanced skill and not standard paramedic practice. Removing TI from standard paramedic practice potentially creates challenges if an SGA device such as the i-gel does not protect a patient’s airway from aspiration or allow adequate ventilation, and clinical staff with enhanced skills are not immediately available to support. This study has highlighted the difficulties paramedics face when managing a soiled airway with an i-gel.

Further research could usefully investigate how paramedics should optimise their management of the patient using an i-gel when the airway is soiled. To our knowledge there is an absence of guidance and training on how best to manage a soiled airway during OHCA with an i-gel and this requires addressing now that TI has been deemphasised in UK standard paramedic practice. Further research could also seek to understand the reasons why patients in the TI group of the trial did worse than patients in the i-gel group of the trial in terms of functional recovery where the allocated airway algorithm was adhered to. However, this would be more challenging to complete now in the UK.

## Conclusion

AIRWAYS-2 study paramedics did not follow their allocated airway management algorithm in 680 (11.7 %) of 5800 patients. There was a higher proportion of deviations in the TI group (399; 14.7 %) compared to the i-gel group (281; 9.1 %). The most frequent reason for deviating from the allocated airway management algorithm in AIRWAYS-2 was obstruction of the patient’s airway by fluid. This occurred in both groups of the AIRWAYS-2 trial, but was more frequent in the i-gel group. The results of this study highlight that paramedics make pragmatic and best interest decisions for patients in this unpredictable and dynamic situation. Paramedic guidelines and training should be enhanced to equip paramedics with the knowledge and skills to best manage a soiled airway in order to maximise patient outcomes during OHCA.

## Funding

10.13039/501100000272National Institute for Health Research: HTA/12/167/102/.

## CRediT authorship contribution statement

**K. Kirby:** Conceptualization, Methodology, Writing – original draft, Writing – review & editing. **M. Lazaroo:** Data curation, Writing – review & editing. **J. Green:** Writing – review & editing. **H. Hall:** Writing – review & editing. **R. Pilbery:** Writing – review & editing. **G.A. Whitley:** Writing – review & editing. **S. Voss:** Conceptualization, Methodology, Writing – review & editing. **J. Benger:** Conceptualization, Methodology, Writing – review & editing, Supervision.

## Declaration of Competing Interest

The authors declare that they have no known competing financial interests or personal relationships that could have appeared to influence the work reported in this paper.
