# Supplementary material for: The reality of advanced airway management during out of hospital cardiac arrest; why did paramedics deviate from their allocated airway management strategy during the AIRWAYS-2 randomised trial?
Source: Resusc Plus. 2023 Feb 18;13:100365. doi: 10.1016/j.resplu.2023.100365 (PMC9969270; doi:10.1016/j.resplu.2023.100365)
Supplement: Supplementary data 1 [file mmc1.docx]

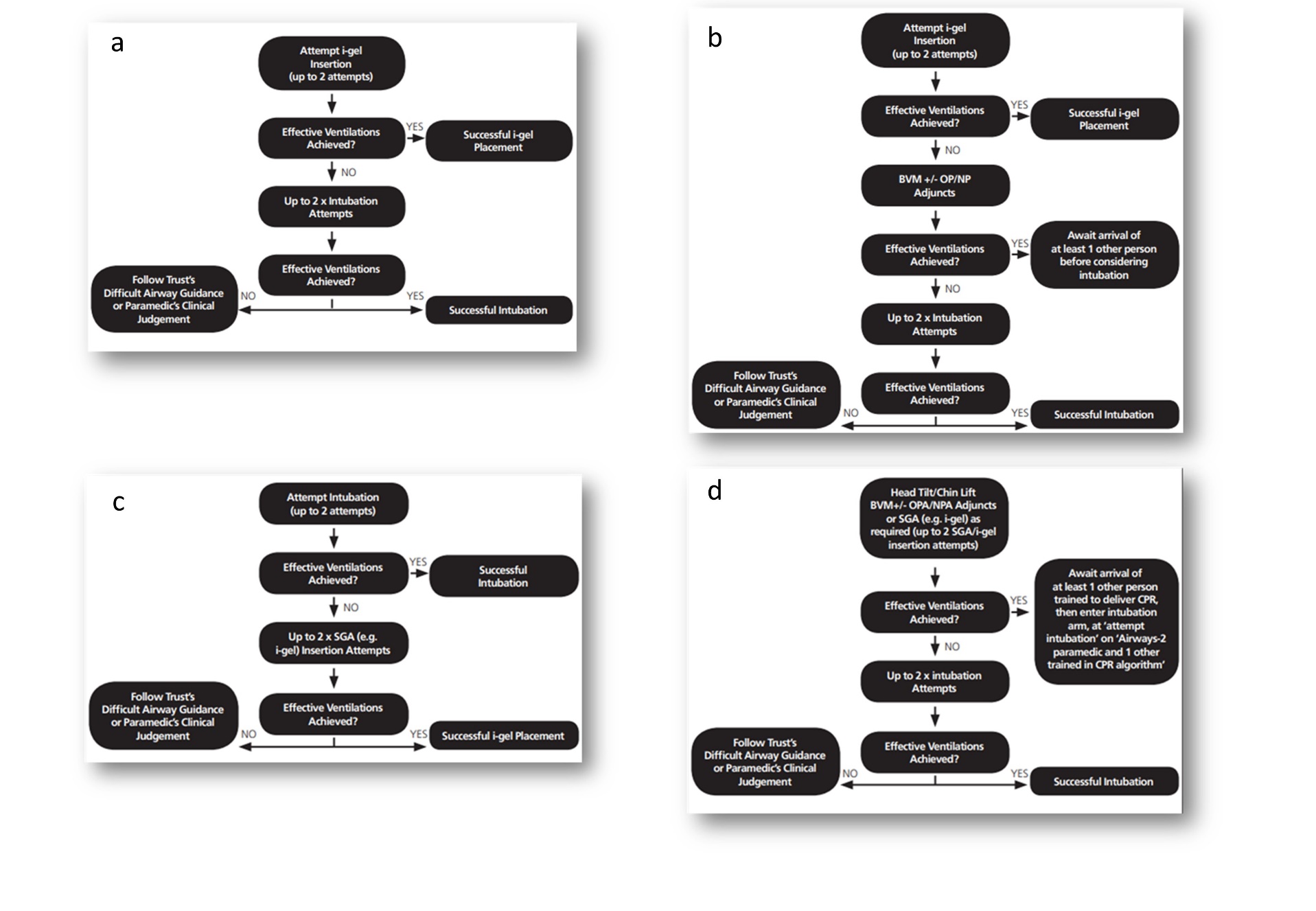


Supplementary Figure 1: AIRWAYS-2 initial airway management algorithms (a) i-gel Airways-2 paramedic and at least one other person trained in CPR. (b) i-gel Solo Airways-2 Paramedic Response. (c) Intubation Airways-2 paramedic and at least one other person trained in CPR. (d) Intubation Solo Airways-2 Paramedic Response^5^. (Permission to reproduce)
